# Lessons from the COVID-19 pandemic: remote coaching in bariatric surgery

**DOI:** 10.1007/s00423-022-02612-7

**Published:** 2022-07-19

**Authors:** Mario Musella, Gennaro Martines, Giovanna Berardi, Arcangelo Picciariello, Giuseppe Trigiante, Antonio Vitiello

**Affiliations:** 1 Advanced Biomedical Sciences Department, Naples “Federico II” University, AOU “Federico II” - Via S. Pansini 5, 80131 Naples, Italy; 2 General Surgery Unit “M. Rubino” Azienda Ospedaliero Universitaria Policlinico, 70124 Bari, Italy

**Keywords:** Education, Training, Bariatric surgery, Sleeve Gastrectomy, Telementoring, Remote coaching, COVID-19

## Abstract

**Background:**

The development of fast internet connection has stimulated different types of video-assisted teaching programs. However, a remote mentoring with the proctor not on site has never been reported in bariatric surgery. We described our experiences with remote telementoring for laparoscopic sleeve gastrectomy.

**Methods:**

A qualified general surgeon at the beginning of his bariatric practice performed a series of 8 laparoscopic sleeve gastrectomies (LSG) while tutored by an experienced bariatric surgeon connected from a different city through a specific videoconferencing platform. Data on demographics at baseline, operative time, hospital stay, intraoperative early, and late complications were collected.

**Results:**

Mean age and BMI of patients were 36.9 ± 9.6 years old and 41.8 ± 1.7 kg/m^2^. All procedures were carried out without conversion to open or complications. Mean operative time was 112.4 ± 21.9 min while the hospital stay was 3.5 ± 0.5 days. Operative time significantly decreased after the fourth operation.

**Conclusions:**

Remote coaching appears to be possible and safe for LSG.

## Introduction

The laparoscopic sleeve gastrectomy (LSG) is the most frequently performed bariatric procedure [1] and the reason of this worldwide diffusion is its laparoscopic feasibility.

Even if the surgical technique of LSG does not require stitching or anatomical rearrangements of the gastrointestinal tract, an appropriate learning curve (LC) is mandatory to reduce perioperative complications and improve results[2]. Fifty to one hundred cases are necessary for a newly trained surgeon to reach the plateau of LC [3]. Even if general laparoscopic surgeons probably need a shorter training, teaching is usually performed onsite with the physical presence of the mentor in the operating room. Indeed, bariatric surgery requires specific skills, and, especially in case of an intraoperative complication, takeover from an expert surgeon may be necessary.

During the COVID-19 pandemic, all non-urgent/non-oncologic outpatient activities and surgical procedures were suddenly interrupted. Bariatric surgery was allowed only under specific guidelines of the Italian Society of Surgery for Obesity (S.I.C.OB.) [4]. Surgical training and proctoring programs went under intense pressure due to the reduction of interventions and the travel limitations imposed by the government. Over the last 15 years, several articles have shown that telementoring is possible [5], but during the COVID-19 pandemic, it became a necessity. Very recently, effectiveness of a teleproctoring program for endoscopic sleeve gastroplasty was proven [6]. However, there is still no report of telementoring for LSG to date. The aim of this study was to describe first worldwide experience with telementoring for laparoscopic sleeve gastrectomy.

## Methods

Before the Sars-Cov-2 pandemic, a mentoring program was scheduled between two university hospitals in different cities (Naples and Bari). The original program provided onsite tutoring with the physical presence of an expert surgeon in the operating room with the mentee. Due to travel restrictions, this schedule could not be followed during the epidemic of COVID-19. Therefore, an experimental telementoring platform was used to allow a remote coaching program.

This platform was intentionally designed for intraoperative surgical mentoring and education. Despite that it does not provide augmented reality or telestration, it can function over low-speed internet connections and is compatible with almost any electronic device having a camera component. While the scope pointed at the target of interest, both the coach and mentee can simultaneously view its image in real time on their respective monitors.

**Fig. 1:**
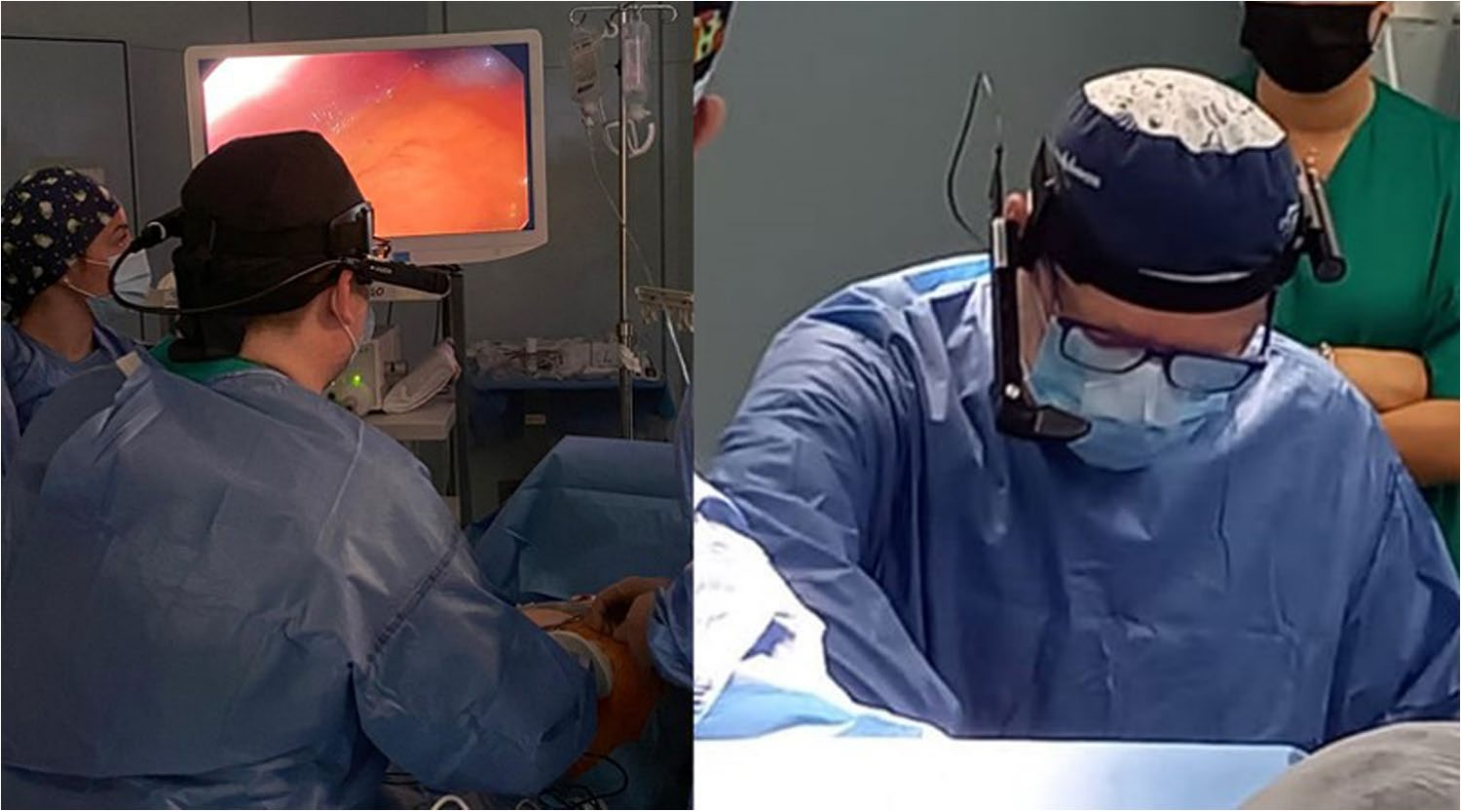
Operating surgeon/mentee wearing earphones and special glasses

The coach/mentor was in his office in Naples, using the platform to watch the operation on his personal computer through an IP-based/internet connection. Audio–video interactions were allowed using earphones and a microphone connected to his pc. The tutored surgeon, while in his operating room in Bari, was wearing a headset providing earphones and a microphone for a real-time verbal interaction, and special glasses, whose screen could show written texts coming from the proctor keyboard (Fig. 1).

The mentee was a general surgeon with prior experience with laparoscopic gastric index procedures (> 50 laparoscopic gastric bandings), but in his learning curve for the laparoscopic sleeve gastrectomy. Data on demographics at baseline, operative time, hospital stay, intraoperative early, and late complications were collected.

All procedures were performed between May and October 2021 and patients signed a written informed consent. The project was approved by the local ethics committee.

### Surgical technique

Surgical technique has been described in detail elsewhere [7], but a brief description is reported below for completeness of the article. For LSG, a five-trocar approach (3 × 12 mm, 2 × 5 mm) was used. The gastrectomy started 4–6 cm from the pylorus over a 38–40 French bougie. Staple line reinforcements or oversewing were not used.

### Statistical analysis

All data were retrospectively collected. Continuous variables were reported as mean ± standard deviation (SD) and as frequencies and proportions for categorical ones. Correlation between experience and operative time was calculated using a linear regression with case number as the independent variable and procedure time as the dependent one. *T*-test was used to compare means. Analysis was performed using SPSS 27.0 (SPSS Inc., Chicago, IL), with statistical significance defined as *P* < 0.05.

## Results

Eight consecutive cases (3 males, 5 females) were successfully performed using a telementoring program between Naples and Bari.

**Fig. 2:**
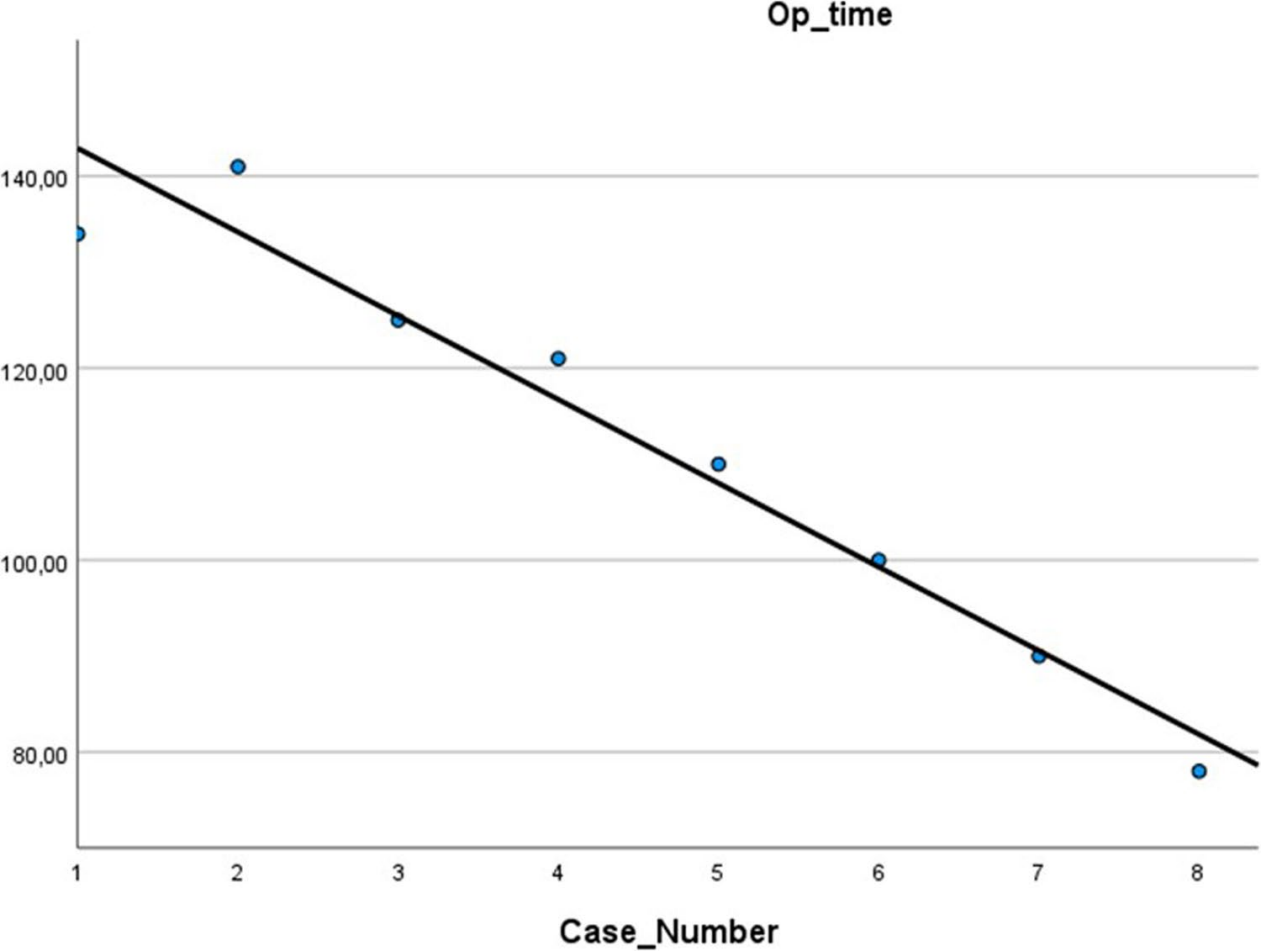
Curve of linear regression between case number and operative time

Mean age and BMI of patients were 36.9 ± 9.6 years old and 41.8 ± 1.7 kg/m^2^, respectively. All procedures were carried out without conversion to open or intraoperative complications. Mean operative time was 112.4 ± 21.9 min while the hospital stay was 3.5 ± 0.5 days. Linear regression showed strong correlation between case number and operative time (*r*-squared = 0.9; *P* < 0.01; Fig. 2).

Comparison of first four cases versus the last four showed a significant reduction of mean operative time (130.3 ± 8.9 vs 94.5 ± 13.7; *P* = 0.005).

## Discussion

In the last years, there has been a large use of videoconferencing programs. In these meetings, an experienced surgeon performs an intervention while connected to a group of colleagues or students in order to show tips and tricks of the procedure.

This approach represents a useful tool to guarantee a continuous exchange of knowledge between experts and it also allows non-expert colleagues to learn from skilled surgeons.

Moreover, an increasing body of evidence demonstrates that a retrospective coaching of a video performance of a learner has a positive impact on surgical proficiency [8].

“Telementoring is a telemedicine technique that involves the remote guidance of a treatment or a procedure where the caregiver has no or limited experience with the featured technique [9].” Therefore, the main difference with other approaches of video-assisted training is that the mentor cannot physically intervene. Subsequently, telementoring has been advocate as a useful tool to provide surgical subspecialty advices [10–12]. Remote mentors can facilitate interventions that would not be performed due to complexity and lack of experience by local surgeons. Real-time verbal interaction is also important in case of an unexpected intraoperative finding or emergency.

In times of robotic surgery, remote wireless connection could also allow surgeons to teach and operate without being in the operating room [13], and nowadays, vast majority of theaters has screens and internet connections allowing video and audio interaction.

As for telemedicine, telementoring was initially ideated and used to help physicians/surgeons in rural areas, but during the pandemic, benefits of this technology have become more evident. Remote interaction, instead of a physical presence, also reduces costs of travels and allows teachers to save time for other activities.

Usefulness of telestration and augmented reality in general and bariatric surgery has recently been demonstrated [14].

Our experience is limited to a restricted number of patients, but it represents an important evidence of remote coaching in bariatric surgery.

Despite that LSG is technically less complex than other bariatric procedures, it takes at least 50–100 cases for a newly trained surgeon to become proficient[15, 16]. In our series, operative time progressively decreased with a significant reduction already after the fourth case.

Perioperative outcomes demonstrated that remote coaching is safe when the mentee is a laparoscopic surgeon who is starting his bariatric practice. Nevertheless, extreme caution should be exercised during dissection of short gastric vessels and gastric fundus mobilization.

During the learning curve of a specific procedure, surgeons with prior experience with other index operations do not always have the chance to have a mentor on site. In many occasions, they rely on their “general experience” in abdominal surgery to manage an intraoperative complication, such as bleedings, perforations, or leaks. The aim of remote coaching is to prevent these complications from happening and to offer prompt and helpful instructions in case they occur.

However, even if all surgeons involved in this study reported high satisfaction, doubts were raised regarding the use of this approach for more complicated bariatric interventions. Indeed, revisional surgery and the gastric bypass procedures may require takeover from a skilled colleague, and in those cases, the expert surgeon should be on site.

### Strength and limitation

Retrospective nature and the absence of a control group are main limitations of this study. Robust statistical analysis was not possible due to the small sample size. However, feasibility of remote coaching has been rarely reported before in bariatric surgery and significant improvements have been demonstrated after few cases.

## Conclusion

Remote coaching of a general surgeon in his learning curve of LSG appears to be possible and safe when the mentee is an experienced laparoscopic surgeon. Larger prospective studies are needed to confirm these findings.
